# Severe Thrombocytopenia Secondary to Severe Iron Deficiency Anemia due to Menorrhagia

**DOI:** 10.1155/crh/3894943

**Published:** 2025-05-18

**Authors:** Tyler E. Russeth, Amanda Luong, Mandi Liu, Mihir Shah, Nicole Desai

**Affiliations:** ^1^Department of Internal Medicine, Temple University Hospital, Philadelphia, Pennsylvania, USA; ^2^Lewis Katz School of Medicine at Temple University, Philadelphia, Pennsylvania, USA

## Abstract

Thrombocytosis is commonly seen in patients with iron deficiency anemia and often normalizes following iron supplementation. Thrombocytopenia with iron deficiency anemia is a less common occurrence that can be seen in severe cases. This phenomenon is well documented in the pediatric population secondary to nutritional deficiency but is underreported in the adult population. Similarly, thrombocytopenia resolves following iron supplementation but the mechanism behind this and why select patients are affected is not well understood. This case report describes a young woman with menorrhagia who was found to have iron deficiency anemia and severe thrombocytopenia with resolution following intravenous iron.

## 1. Introduction/Background

According to the World Health Organization, the estimated prevalence of anemia is 24.3% with the most common cause being iron deficiency anemia (IDA) [1]. The most common underlying etiologies for IDA are menstruation in women and gastrointestinal bleeding [2]. Patients can experience shortness of breath, headaches, fatigue, tachycardia, and in severe cases, hypoxia [2]. The physiologic role of iron in hematopoiesis is well established; however, its impact on platelet production is not fully understood [3]. While thrombocytosis is commonly seen in conjunction with IDA, thrombocytopenia can also be observed. Several case reports and series exist that describe thrombocytopenia and IDA in the pediatric population, but cases in the adult population are infrequent. In this case report, we present a case of thrombocytopenia secondary to IDA in a young woman with menorrhagia with a brief review of currently available literature.

## 2. Case Report

A young woman in her forties with a history of menorrhagia secondary to uterine fibroids, vitamin B12 deficiency, and IDA presented for increased vaginal bleeding associated with worsening fatigue, lightheadedness, and lower abdominal pain. Her last menstrual cycle was 2 weeks prior to presentation when she experienced her normal cycle of 7 days of vaginal bleeding requiring 3-4 pad changes daily. However, she later began to experience vaginal spotting with progressive bleeding and passing of blood clots. Her surgical history included bilateral tubal ligation and cesarean section. Family history included lupus in her mother. She was not actively taking medications but recollected previously taking vitamin B12 and iron supplements. She drank alcohol on social occasions and denied any tobacco or drug use. She ate a normal diet which included meat products. She also consumed three cups of ice daily.

Vital signs were stable with temperature 97.6 F, blood pressure 135/51, heart rate 54, and SpO_2_ 99% on room air. On physical exam, she was well nourished. She had pale conjunctiva without evidence of gingival bleeding or scleral icterus. Lungs were clear to auscultation and cardiac exam was notable for bradycardia without an audible flow murmur. There was mild suprapubic tenderness to palpation without significant hepatosplenomegaly. No petechiae or ecchymoses were seen. Pelvic exam showed scant blood at the cervical os with a dime-sized clot in the vaginal vault. Bimanual examination showed a large, mobile, anteverted uterus with subserosal fibroids without adnexal masses.

Initial serologies demonstrated a hemoglobin of 5.2 g/dL, mean corpuscular volume (MCV) 65.0 fL, and red cell distribution width (RDW) 35.4%. Reticulocyte count was normal but with a depressed reticulocyte index of 0.19. Ferritin was very low at 5 ng/mL. Vitamin B12 and methylmalonic acid were low. Notably, a severe thrombocytopenia of 14 K/mm^3^ was identified (Table 1). Additional iron studies were not obtained prior to red blood cell transfusions.

Further workup for the patient's thrombocytopenia revealed normal liver enzymes without coagulopathy or evidence of hemolysis. Thyroid studies, antinuclear antibody, human immunodeficiency virus, hepatitis C, rheumatoid factor, celiac panel, Epstein–Barr virus, and von-Willebrand factor activity were normal (Table 1). Intrinsic factor blocking antibody was equivocal but with a high parietal cell antibody titer (Table 1). Urinalysis and chest imaging were negative for infection.

Peripheral blood smear showed marked anisocytosis and poikilocytosis with moderate hypochromia and markedly decreased platelets. No platelet clumping and no granulocyte dysplastic changes were seen. There were occasional teardrop and burr cells without hypersegmented neutrophils. Abdominal ultrasound showed hepatomegaly with steatosis without splenomegaly. Pelvic ultrasound revealed an enlarged, heterogenous, leiomyomatous uterus.

**Figure 1:**
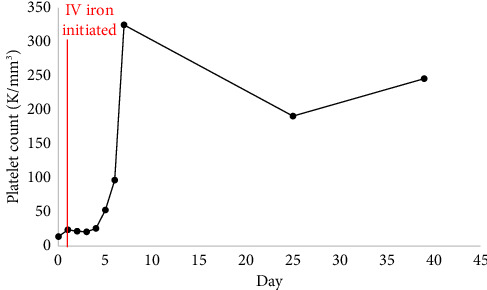
Platelet counts during hospitalization and following discharge. Day 0 represents the day of admission. The patients received one unit of platelets on Day 0. A total of 125 mg IV sodium ferric gluconate was administered daily on hospital Days 1–4 with discharge on Day 5. An additional five 200 mg IV iron sucrose infusions were given over Days 8–22.

She received two red blood cell transfusions for a goal hemoglobin of greater than 7 g/dL with symptomatic improvement [4]. One unit of platelets was transfused on day of admission (Day 0) due to active bleeding [5]. Repeat platelet count indicated an adequate response. She was started on 10 mg medroxyprogesterone acetate twice daily with interval improvement in vaginal bleeding. Due to the severity of her IDA and active blood loss, administration of 125 mg intravenous (IV) sodium ferric gluconate daily was started on Day 1 of hospitalization. Oral iron supplementation was not chosen due to concerns that blood loss would exceed oral iron absorptive capacity [6]. After 4 days of IV iron, her platelet counts markedly improved (Figure 1). A bone marrow biopsy was not pursued due to rapid clinical improvement. The patient was discharged on Day 5 with oral 325 mg ferrous sulfate every other day and oral 1000 mcg vitamin B12 daily.

The patient followed up at the infusion clinic for an additional five 200 mg iron sucrose infusions over the course of 2 weeks. Her hemoglobin and platelet counts remained stable with an improved ferritin in the outpatient setting (Table 2). It was determined that the patient's thrombocytopenia was secondary to IDA in the setting of menorrhagia.

## 3. Discussion and Literature Review

Classically, IDA is associated with normal platelet counts or thrombocytosis with has an estimated prevalence of 4.4% [7]. Patient's platelet levels typically normalize following oral or IV iron repletion [7, 8]. On the contrary, thrombocytopenia (platelet counts less than 150 × 10^9^/L) can also be observed. This phenomenon is commonly seen in the pediatric population and can affect upwards of 28.3% of patients with IDA [9]. Many cases are a result of nutritional deficiency from excessive cow's milk consumption and platelet counts recover following administration of iron [9, 10]. In the adult population, thrombocytopenia is only seen in roughly 2.1%–2.3% of the patients with IDA and predominantly affects women in their third decade of life [11, 12].

Several case reports exist describing this in young women with menorrhagia similar to our patient [13–16]. All women presented with severe anemia necessitating transfusion and iron studies were consistent with IDA. All received oral or IV iron supplementation with marked improvement in platelet counts. However, the patient described in Berger et al. did not see an immediate response and required platelet transfusions due to continued menorrhagia [16]. Verma et al. and Huscenot et al. represent the largest case series of women with IDA and thrombocytopenia [17, 18]. Many of their patients were young women with menorrhagia who achieved normal platelet levels within 4-5 days following administration of iron.

The mechanism of IDA and thrombocytopenia remains poorly understood. Thrombocytosis and IDA occur due to a preferential shift toward formation of megakaryocyte-erythroid progenitors and megakaryopoiesis [3]. Patients with IDA and thrombocytopenia often present with severe anemia; thus, it is hypothesized that the erythropoiesis pathway takes precedent resulting in decreased platelet production [3]. Alternatively, iron is known to be an essential component to thrombopoiesis and perhaps, this process can no longer occur after reaching a certain threshold of iron deficiency.

Our case contributes further to the body of evidence linking thrombocytopenia to IDA. Like other cases, our patient presented with acute blood loss due to abnormal uterine bleeding. Her initial labs demonstrated severe IDA and thrombocytopenia. Workup for the thrombocytopenia excluded causes such as infections, autoimmune disease, and sequestration (Table 1). Idiopathic thrombocytopenia purpura (ITP) was ruled out with an appropriate response to platelet transfusion. Platelet consumption due to menorrhagia was likely contributory but it was not believed to be the primary driver. If acute blood loss anemia was the primary driver, hemodynamic instability would be expected. Vitamin B12 deficiency and pernicious anemia was heavily considered as the underlying etiology but felt to be less likely as there was no evidence of hemolysis or schistocytes seen on blood smear which is commonly observed in these cases [19].

## 4. Conclusion

Iron deficiency-related thrombocytopenia is a less common diagnosis. Prompt recognition of this clinical scenario can prevent further costly and invasive procedures such as bone marrow biopsy. In addition, overlooking this diagnosis may lead to putative treatment for ITP with steroids and IV immunoglobulins.

## Figures and Tables

**Table 1:** Initial laboratory serologies at presentation with their corresponding reference ranges.

Variable (units)	Value (reference range)
Hemoglobin (g/dL)	5.2 (12.3–15.3)
Hematocrit (%)	18.3 (35.9–44.6)
Mean corpuscular volume (fL)	65 (80.0–96.0)
Red cell distribution width (%)	35.4 (11.5–14.5)
Platelet count (K/mm^3^)	14 (150–450)
White blood cells (K/mm^3^)	5.9 (4.0–11.0)
Total bilirubin (mg/dL)	0.5 (0.0–1.0)
Aspartate aminotransferase (U/L)	8 (15–37)
Alanine aminotransferase (U/L)	13 (16–61)
Ferritin (ng/mL)	5 (8–388)
Vitamin B12 (pg/mL)	186 (211–911)
Methylmalonic acid (nmol/L)	83 (87–318)
Folate (ng/mL)	6.47 (5.38–24.00)
PTT (seconds)	28.2 (25.1–36.5)
PT (seconds)	11.5 (9.4–13.1)
INR	1.0
Reticulocyte count (%)	1.1 (0.5–1.5)
Haptoglobin (mg/dL)	114 (30–200)
Fibrinogen (mg/dL)	193 (200–393)
Lactate dehydrogenase (U/L)	102 (84–246)
Thyroid stimulating hormone (m[iU]/L)	0.799 (0.400–4.500)
von-Willebrand activity (%)	127 (49–163)
Intrinsic factor blocking antibody	Equivocal
Parietal cell antibody (titer)	> 1:640 (< 1:20)

*Note:* All values are expressed as actual value with corresponding reference range.

**Table 2:** Complete blood count and iron studies on the day of admission and Day 40 in the outpatient setting.

Variable (units)	Reference range	Day 0 (admission)	Day 40
Hemoglobin (g/dL)	12.3–15.3	5.2	10.4
Hematocrit (%)	35.9–44.6	18.3	34.1
Mean corpuscular volume (fL)	80–96	65	87.9
Red cell distribution width (%)	11.5–14.5	35.4	22.2
Platelet count (K/mm^3^)	150–450	14	246
Ferritin (ng/mL)	8–388	5	76
Total iron (mcg/dL)	40–190	—	29
Total iron binding capacity (mcg/dL)	250–450	—	387
% Iron saturation (%)	16–45	—	7

*Note:* All values are expressed as actual value with corresponding reference range. Further iron studies were not collected on day of admission prior to red blood cell transfusion.

## Data Availability

The data that support the findings of this study are available within the article.
